# New Orleans—Climatic, Hygienic, Medical

**Published:** 1873-08

**Authors:** 


### EDITORIAL AND MISCELLANEOUS.

### NEW ORLEANS—CLIMATIC, HYGIENIC, MEDICAL.

The Senior Editor of this JOURNAL, in his recent visit to the meeting of the American Medical Association, at St. Louis, took the circuitous but most attractive route, (to one who has not before traveled it,) by way of New Orleans, and takes great pleasure, in accordance with the spirit and principle of this JOURNAL since his association with it, in presenting some observations in connection with that politically down-trodden, but still most interesting and important medical centre.

New Orleans, with a population of two hundred thousand, is situated at the mouth of the largest river, and is the port of the most extensive and fertile valley in the world.

The winter and spring climate (the period devoted to medical instruction) is delightful and salubrious.

The severity of winter and the excessive heat of summer are moderated by the Gulf of Mexico and the large salt lakes which surround New Orleans, and render its climate insular.

Owing to the cool gulf breezes, and wide streets, and the great space along the banks of the Mississippi occupied by the city, the heat of summer is actually less than in New York and Philadelphia; and, as a consequence, sun-strokes are far less frequent than in the great Northern and Western cities.

The winter and spring climate is so uniform and mild that pneumonia, pleurisy, and rheumatism and inflammatory affections are almost unknown. No healthier body of men are to be found upon the continent, than the medical students that assemble in the winter and spring in New Orleans. The change to this mild, sub-tropical climate is especially delightful and beneficial to the natives of colder and more elevated latitudes. Medical students who are suffering with pulmonary complaints, or who dread the cold, damp climates of Baltimore, Philadelphia, New York, Louisville, Nashville, Cincinnati, Chicago, and

St. Louis, would find it advantageous to prosecute their medical studies in the mild and healthy climate of New Orleans.

The resident population of New Orleans are noted for the enjoyment of good health, and, as a general rule, attain to a good old age. No city in the United States can boast of a larger number of healthy and fine looking children. The climate is so mild as to permit of the finest ventilation, and children are enabled to spend the greatest time in the open air.

Whilst the resident population of New Orleans is estimated at two hundred thousand, at least from one hundred thousand to one hundred and fifty thousand strangers, emigrants, and seamen, from all the States of the Union, and from every European nation, annually pass through this great emporium of commerce.

The clinical advantages afforded by New Orleans to medical students are unsurpassed, and perhaps unequaled, upon this continent. For the study of southern diseases, the facilities afforded by the great Charity Hospital are unequaled in this country or in any other.

Situated in the heart of this great city, the doors of this noble institution are ever open to the sick of every State—North, East, West, and South—and of every nation upon the face of the globe. More than one hundred thousand dollars are annually expended by the State of Louisiana in the support of the sick patients in the Charity Hospital, although the native Louisianians who are borne to its well ventilated and carefully served wards constitute not more than from one-seventh to one-tenth of the whole number of patients annually treated. Thus, during the past year, of 5,541 patients, 3,250 were foreigners, 1,660 natives of various Northern and Western and Southern States, and only 631 natives of Louisiana. Amongst foreign nations, Ireland furnished, during the past year, 1,489; Germany, 730; England, 211; France, 252; and even China contributed 44 patients. Amongst the United States, New York gave 197; Virginia, 264; Kentucky, 164; Pennsylvania, 82; Missouri, 86; Alabama, 84; North Carolina, 87; Tennessee, 106; Mississippi, 123; Maryland, 77, and even Georgia was represented by 52 sick and destitute.

Every State in the great valley of the Mississippi should contribute to the support of the Charity Hospital of New Orleans.

During the thirteen years preceding 1843, the Charity Hospital received into its wards for comfort and relief, when aid could not be expected elsewhere, 59,621 patients; in 1850, 18,423 patients were admitted; and during the past thirty years, there have been admitted, treated, and cared for, no less than 295,786 patients, a number equal to one-fourth of the entire population of the great State of Georgia.

This gigantic mass of disease is rendered available for clinical instruction.

The act establishing the University of Louisiana gives the Professors of the Medical Department the use of the Charity Hospital as a school of practical instruction.

The Charity Hospital contains nearly seven hundred beds, and received, during the last year, 6,637 patients. Its advantages for professional study are unequaled by any similar institution in this country. The medical, surgical, and obstetric wards are visited daily, by the respective professors in charge, from 8 to 10 o'clock; at which time all the students are expected to attend and familiarize themselves, *at the bed-side of the patients*, with the diagnosis and treatment of all forms of injury and disease. Special instruction is given to the candidates for graduation who are, for this purpose, divided by the Dean of the faculty into classes, and assigned to the respective professors in charge of wards in the Hospital. The classes thus formed interchange courses, so as to enable the candidates to enjoy equal advantages.

Thoroughly competent chiefs of clinics aid the clinical teachers in developing to its full extent this system of instruction. Students who are not candidates for graduation make the daily rounds with any of the clinical teachers.

The regular lectures at the hospital—on Clinical Medicine, by Professors Bemiss and Joseph Jones; Surgery, by Professors Richardson and Logan; Diseases of Women and Children, by Professor Hawthorne; and Special Pathological Anatomy, by Professor Chaillé—are delivered in the Amphitheatre of the Charity Hospital, on Monday, Wednesday, Thursday, and Saturday, from 10 to 12 o'clock A.M.

In addition to the regular systematic didactic lectures given by the several professors in the central building of the University, situated on Common street, between Baronne and

Dryade streets, there is attached to each chair a practical department in which students can observe and verify for themselves the facts inculcated and discussed in the lecture-room.

The professor of Chemistry and Clinical Medicine, Dr. Joseph Jones, has, during the past four years, founded, by his own efforts and at his own expense, a Practical Laboratory of Medical Chemistry, Pharmacy, and Toxicology, in which the students are furnished with chemical re-agents, and are instructed in the modes of experiment and analysis, which are necessary for a practical knowledge and application of medical physics and chemistry.

The action of poisons and medicines are illustrated by experiments upon animals, and the students are furnished with the necessary apparatus and tests for the detection and analysis of poisons.

Special instruction is given upon the chemical constitution, physiological and and pathological relations, and methods of analysis of blood and urine. The students are furnished with microscopes, and instructed in the appearance and mode of examination of diseased structures and secretions, blood, and urine. The private library of Professor Joseph Jones, numbering over three thousand valuable works, on medicine and the related sciences, is attached to his Practical Laboratory.

The means for the illustration of the systematic didactic lectures in the University, consist in models in wood, wax, plaster, and papeir mache of rare and beautiful workmanship, executed in London, Paris, and Florence, expressly for the University; a complete series of specimens of materia medica; surgical instruments and appliances; specimens in morbid anatomy; and an entire set of chemical and philosophical apparatus.

The faculty have established a free library and reading-room for the use of students and physicians, in the spacious hall in the east wing of the University.

The Medical Department of the University of Louisiana, since its foundation in 1834, has had 6,860 matriculates, and 1,690 graduates.

The Charity Hospital furnishes an abundance of dissecting material, and the dissecting rooms are large and well ventilated and lighted.

The buildings are probably as well adapted to their purpose as those of any medical institution in the United States, and the lecture-rooms, are capable of seating, each, about five hundred students.

It will afford us pleasure in the future to chronicle the full relief of this people from the iron heel of the oppressor, and an entire restoration of the former prosperity of the city, in a medical as well as commercial point of view.

